# Screening of differentially expressed microRNAs of essential hypertension in Uyghur population

**DOI:** 10.1186/s12944-019-1028-1

**Published:** 2019-04-11

**Authors:** Yuanzheng Ye, Jianzhong Yang, Wenkui Lv, Yanmei Lu, Ling Zhang, Ying Zhang, Zulifeiya Musha, Ping Fan, Bin Yang, Xianhui Zhou, Baopeng Tang

**Affiliations:** Heart Center, the First Affiliated Hospital of Xinjiang Medical University, No.137 Liyushan South Road, Urumqi, Xinjiang, 830054 China

**Keywords:** Essential hypertension, Uyghur population, microRNA, Biomarker

## Abstract

**Background:**

Essential hypertension can cause many kinds of cardiovascular diseases. The pathogenesis of essential hypertension is very complex, and the mechanism is still unclear. The microRNAs have been identified as novel biomarkers for pre-diagnosis and prognosis of hypertension. However, the kinds of microRNAs that can be used as specific biomarkers for hypertension are unknown.

**Methods and results:**

Plasma samples were isolated from Uyghur subjects with essential hypertension and the healthy individuals. Microarray was used to identify differentially expressed microRNAs. The microarray data were clustered and annotated with online software. The target genes of differentially expressed microRNAs were also analyzed. The microarray results were further verified by quantitative real-time PCR. We identified 257 microRNAs that were differentially expressed between patients with essential hypertension and the healthy individuals. These microRNAs had a total of 6580 target genes. The 47 microRNAs that had target genes, including 24 up-regulated and 23 down-regulated microRNAs, were further screened out to construct a reference set of potential microRNA biomarkers. Most of the 47 microRNAs were located at chromosome 19 (40 microRNAs) and chromosome 1 (45 microRNAs). Their target genes were mainly enriched in metal ion binding, transcription regulation, cell adhesion and junction, indicating that these candidate microRNAs may regulate mineral ion binding and cell communication process of essential hypertension. The quantitative real-time PCR results of miR-198 and miR-1183 (which were the two most significantly up-regulated microRNAs by microarray), and, miR-30e-5p and miR-144-3p (which were the two most significantly down-regulated microRNAs by microarray) were consistent with the microarray results.

**Conclusions:**

A reference set of potential microRNA biomarkers that may be involved in essential hypertension is constructed. Our study may provide experimental evidence for further studying the mechanism of essential hypertension.

**Electronic supplementary material:**

The online version of this article (10.1186/s12944-019-1028-1) contains supplementary material, which is available to authorized users.

## Introduction

Essential hypertension is not only one of the main causes but also one of the clinical manifestations of cardiovascular diseases. It is a highly complex disease that can be induced by both environmental and genetic factors [1, 2]. The mechanism of essential hypertension is still unclear. There are still no precise pre-diagnosis methods or efficient treatment options for essential hypertension [2–4]. Thus, it is necessary to identify specific markers for essential hypertension.

MicroRNA is a kind of small non-coding RNA with about 20 bp in length that can regulate gene expression at the post-transcriptional level [4–6]. Previous studies demonstrate that microRNAs can be used as diagnostic biomarkers for hypertension and cardiovascular diseases [2, 4]. The abnormal expression of microRNAs in tissues and body fluids has also been reported [2, 4, 6]. It is also found that there were 27 microRNAs differentially expressed in patients with essential hypertension and that the relationship between hmcv-miR-UL122 and hypertension was identified [7]. In addition, the increased expression of miR-505 may play important roles in essential hypertension [8]. Tissue-based studies also illustrate that several microRNAs play remarkable roles in hypertension, such as miR-181a [1, 9], miR-1, miR-21, miR-9 and miR-126 [10, 11].

MicroRNA array and quantitative PCR are two main techniques for detection and examination of microRNAs [2–4, 6]. The microRNA profile of hypertension has been investigated. However, different microRNA biomarker sets were obtained due to the diversity of experiment methods and research subjects [1–4]. More microRNA markers for essential hypertension remain to be identified. In this study, we screened the microRNA profile of essential hypertension in Uyghur population. Our study may provide experimental evidence for investigating the possible role and mechanism of microRNAs in essential hypertension.

## Materials and methods

### Sample collection and processing

Eight Uyghur subjects aged between 30 and 40 years old were recruited. Four subjects, including 2 males and 2 females, with untreated essential hypertension were included in the hypertension group. Inclusion criteria: 1) blood pressure should meet one of the following criteria: Sitting blood pressure was higher than or equal to 140/90 mill Hg (1 mmHg = 0.133 kPa) measured by Mercury sphygmomanometer; and/or, average daily blood pressure was higher than or equal to 135/85 mill Hg by 24-h ambulatory blood pressure monitor; 2) patients diagnosed with essential hypertension for the first time; 3) patients were untreated. Exclusion criteria: 1) patients with secondary hypertension; 2) patients with cardiac function III, IV, coronary heart disease, severe pulmonary insufficiency, rheumatic diseases, diabetes, endocrine disorders, severe liver or kidney dysfunction, stroke, peripheral arterial disease, or, mental illness; 3) pregnant or lactating women. Four age and gender matched healthy individuals with normal blood pressure (2 males and 2 females) were included in the control group. Healthy subjects did not smoke and had no history of cancer, diabetes, kidney failure, stroke or peripheral arterial disease. For verification of microRNA expression, another 15 patients with essential hypertension (8 males and 7 females) and 15 healthy individuals (4 males and 11 females) were included. The inclusion and exclusion criteria were same as above.

All subjects were fasted before sampling and sampled at the same time in the morning. They were asked to sit still for 10 min before blood collection. Totally, 3 mL of peripheral blood was collected from each subject and the plasma was then isolated.

All subjects or their families signed the informed consent. The study was approved by Ethical committee of Xinjiang Medical University.

### RNA extraction and miRNA microarray analysis

Total RNAs were extracted from plasma using mirVana PARIS kit (Ambion-1556) and quantified by NanoDrop ND-2000 (Thermo Scientific). RNA quality was checked by Bioanalyzer 2100 (Agilent, G2939A). MicroRNA expression was detected by Agilent Human miRNA Microarray (Agilent, 8*60 K, ID070156). RNAs were hybridized with the array in hybridization oven (Agilent, G2545A). Then, array signals were scanned by Agilent Scanner (Agilent, G2505C). All processes were performed in accordance with the standard guidelines provided by the manufacturer.

The signals were then analyzed by Feature Extraction software (version10.7.1.1, Agilent Technologies). Raw data was obtained, and normalized with the quartile algorithm using Genespring software (version 12.5; Agilent Technologies) to calculate the microRNA expression. MicroRNAs with fold changes greater than two and a *p*-value below 0.05 were defined as differentially expressed.

### MicroRNA annotation

MicroRNAs were clustered using the Hierarchical Clustering method. The chromosome loci of microRNAs were annotated by miRBase (http://www.mirbase.org/) and HGNC (Hugo Gene Nomenclature Committee) (http://www.genenames.org/). Cytogenetic locations of microRNAs and OMIM (Online Mendelian Inheritance in Man) record of essential hypertension were retrieved from OMIM database (http://www.omim.org/).

### Target gene prediction, GO annotation and pathway analysis

Target genes of microRNAs were predicted using three databases of Targetscan, (http://www.targetscan.org/), microRNAorg, (http://www.microrna.org/), and pita, (https://genie.weizmann.ac.il/pubs/mir07/), respectively. The resulted genes shared by three databases were selected and then analyzed using Gene Ontology (GO) and KEGG (Kyoto Encyclopedia of Genes and Genomes) pathway. Furthermore, genes were clustered according to the annotation information by DAVID analysis tool (https://david.ncifcrf.gov/).

### Quantitative real-time PCR

Quantitative real-time PCR (qPCR) was conducted to verify microRNA expression. MicroRNAs were extracted using QIAzol Lysis Reagent (QIAGEN, 5346994), miRNeasy Serum/Plasma Spike-In Control (QIAGEN, 219610) and miRNeasy Serum/Plasma Kit (QIAGEN,217184). cDNA was prepared using miScript II RT Kit (QIAGEN, 218161). MicroRNA expression was detected using miScript SYBR Green PCR Kit (QIAGEN, 218073) on ABI7500 (ABI). Four pairs of Primers were used in qPCR assays, including hsa-miR-1183 (CD201–0149, TIANGEN BIOTECH), hsa-miR-198 (CD201–0085, TIANGEN BIOTECH), hsa-miR-30e-5p (CD201–0340, TIANGEN BIOTECH) and hsa-miR-144-3p (CD201–0011, TIANGEN BIOTECH). miRNA39 was used for normalization in the qRT-PCR assays. The relative gene expression was calculated with 2^-ΔΔCt^ method.

### Statistical analysis

Prism statistical software (GraphPad Software Inc) was used for data analysis. Difference in microRNA expression between essential hypertension group and control group was analyzed by student t-test. A value of *p* ≤ 0.05 was considered to be statistically significant.

## Results

### The expression profile of microRNAs in the essential hypertension group

**Fig. 1:**
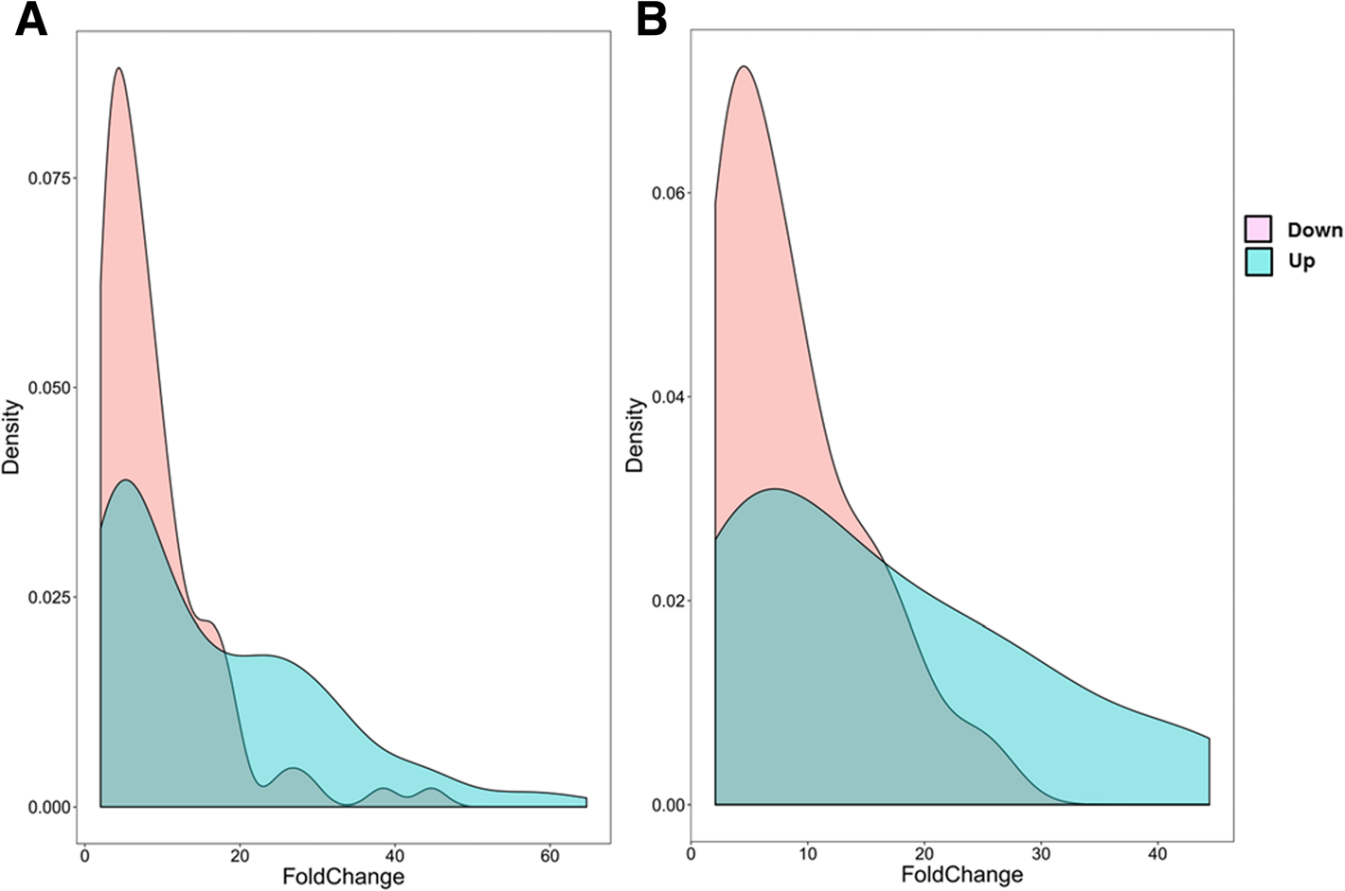
Fold change distribution of differentially expressed microRNA. **a** The left distribution chart illustrates fold changes of all differentially expressed microRNAs. **b** The right chart shows fold change of differentially expressed microRNAs that possess target genes. Pink represents the down-regulated microRNAs. Blue represents the up-regulated miRNAs

**Fig. 2:**
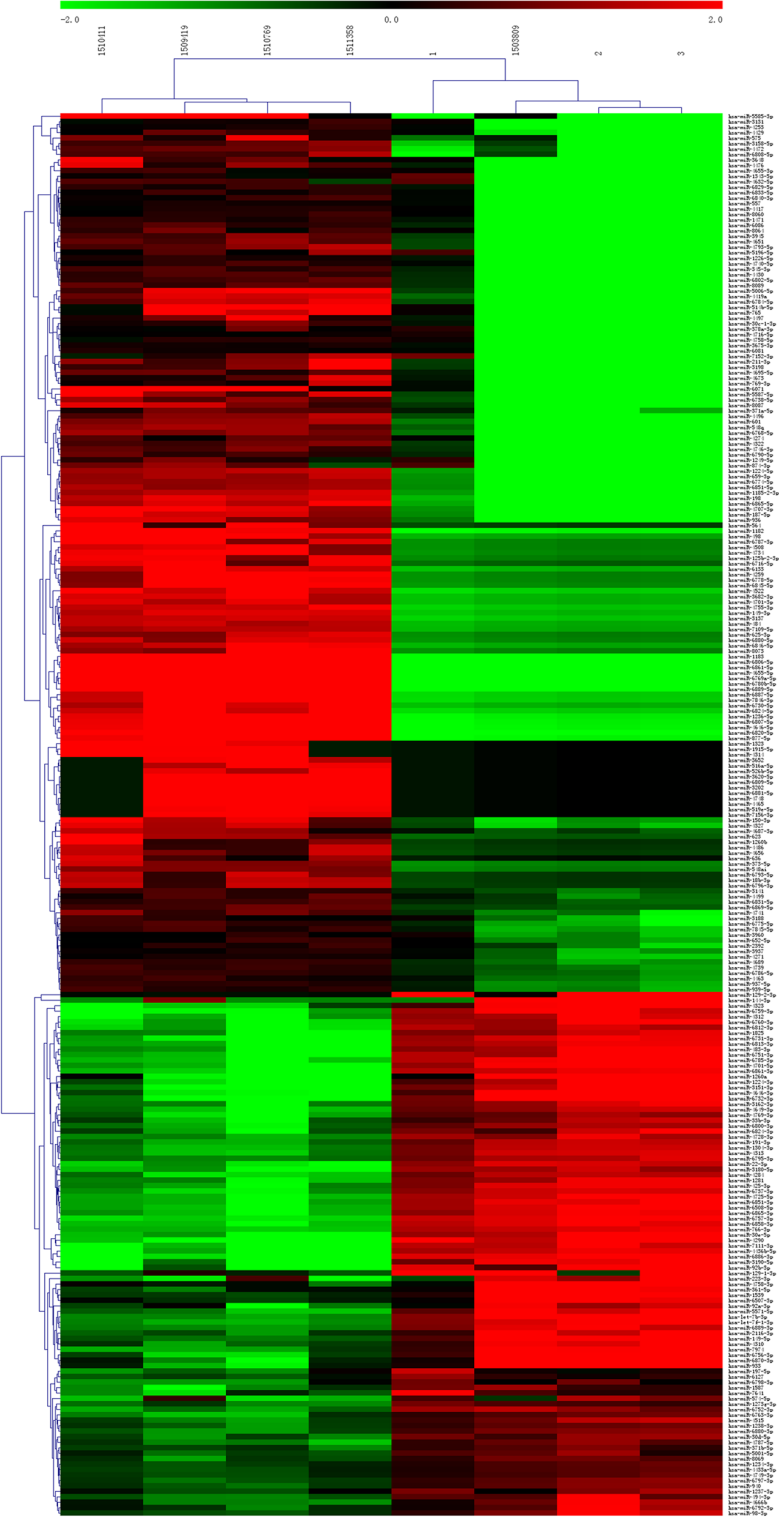
Hierarchical clustering analysis of circulating microRNA expression data from samples of essential hypertension patients (*n* = 4) and the healthy (*n* = 4). MicroRNA expression data are illustrated by the heat map after cluster analysis. X-axis represents sample names. Y-axis represents micro-RNA names. Each lane indicates microRNA expression. The left our lanes represents the microRNA expression in the essential hypertension group and the right four lanes represents the microRNA expression in the control group. Color bar shows the relative expression. Down-regulated microRNAs are shown in green and up-regulated microRNAs are shown in red

Totally, 257 microRNAs that showed differential expression between patients with essential hypertension and the control group were obtained (Table 1). The number of up-regulated microRNAs (161) was higher than that of down-regulated microRNAs (96) (Table 1). Furthermore, fold change distribution of the differentially expressed microRNAs showed distinct pattern. Up-regulated microRNAs changed much more than the down-regulated microRNAs (Fig. 1). The change of hsa-miR-1183 expression (up-regulated, fold change = 44.41) and hsa-miR-30e-5p expression (down-regulated, fold change = 24) were the most significant (Table 2). Cluster analysis classified samples into separate groups based on the microRNA expression in each sample. The microRNA expression of patients in the essential hypertension group was obviously different from that of healthy controls (Fig. 2). Overall, nearly 2/3 microRNAs showed higher level in the essential hypertension samples (Fig. 2).

**Table 1:** Number of differentially expressed microRNA

Classification	Total	UP	DOWN
Differentially expressed miRNA	257	161	96
Differentially expressed miRNA that possess target genes	47	24	23

**Table 2:** List of screened differentially expressed microRNAs

Up-regulated miRNA	*p*-value	Fold change	Down-regulated miRNA	*P*-value	Fold Change
**hsa-miR-1183**	1.07E-07	44.41	**hsa-miR-30e-5p**	0.001839	24.00
**hsa-miR-198**	3.88E-04	42.16	**hsa-miR-144-3p**	0.042303	19.41
hsa-miR-936	0.001909	35.16	hsa-miR-92b-3p	0.001416	17.29
hsa-miR-601	0.001846	30.83	hsa-miR-483-3p	0.002414	15.37
hsa-miR-1224-5p	5.42E-04	29.21	hsa-miR-1260a	0.006716	14.46
hsa-miR-659-3p	7.51E-04	25.28	hsa-miR-1224-3p	0.002259	13.07
hsa-miR-1182	3.07E-05	23.79	hsa-miR-129-2-3p	0.026973	9.70
hsa-miR-557	0.020267	22.84	hsa-miR-1825	9.39E-04	9.28
hsa-miR-877-5p	9.32E-08	19.28	hsa-miR-22-3p	1.44E-05	9.04
hsa-miR-575	0.045361	17.53	hsa-miR-766-3p	3.21E-07	8.89
hsa-miR-371a-5p	0.03657	14.30	hsa-miR-933	0.007722	7.57
hsa-miR-765	0.007593	13.26	hsa-miR-1281	1.49E-04	7.36
hsa-miR-874-3p	0.029926	12.98	hsa-miR-149-5p	6.17E-04	6.02
hsa-miR-498	8.55E-05	12.58	hsa-miR-223-3p	0.038506	4.91
hsa-miR-564	0.035705	9.26	hsa-miR-361-5p	0.012961	4.29
hsa-miR-484	2.52E-08	7.34	hsa-miR-92a-3p	0.016414	4.19
hsa-miR-769-3p	0.013364	5.92	hsa-miR-494-3p	0.002235	3.56
hsa-miR-623	0.004244	5.07	hsa-miR-30d-5p	4.70E-04	3.38
hsa-miR-378a-3p	0.031318	5.02	hsa-miR-574-5p	0.036495	3.19
hsa-miR-1323	0.040302	3.15	hsa-miR-1238-3p	6.79E-04	2.68
hsa-miR-516a-5p	0.040504	2.53	hsa-miR-940	5.81E-04	2.64
hsa-miR-526b-5p	0.041922	2.48	hsa-miR-1237-3p	0.012315	2.23
hsa-miR-939-5p	0.003595	2.42	hsa-miR-1234-3p	3.41E-04	2.09
hsa-miR-636	0.029636	2.08	–	–	–

Four microRNAs of interest are shown in bold. All microRNAs screened above have a fold change of at least 2 and their target genes could be searched in database

### Target gene identification and filteration of candidate microRNA markers

**Fig. 3:**
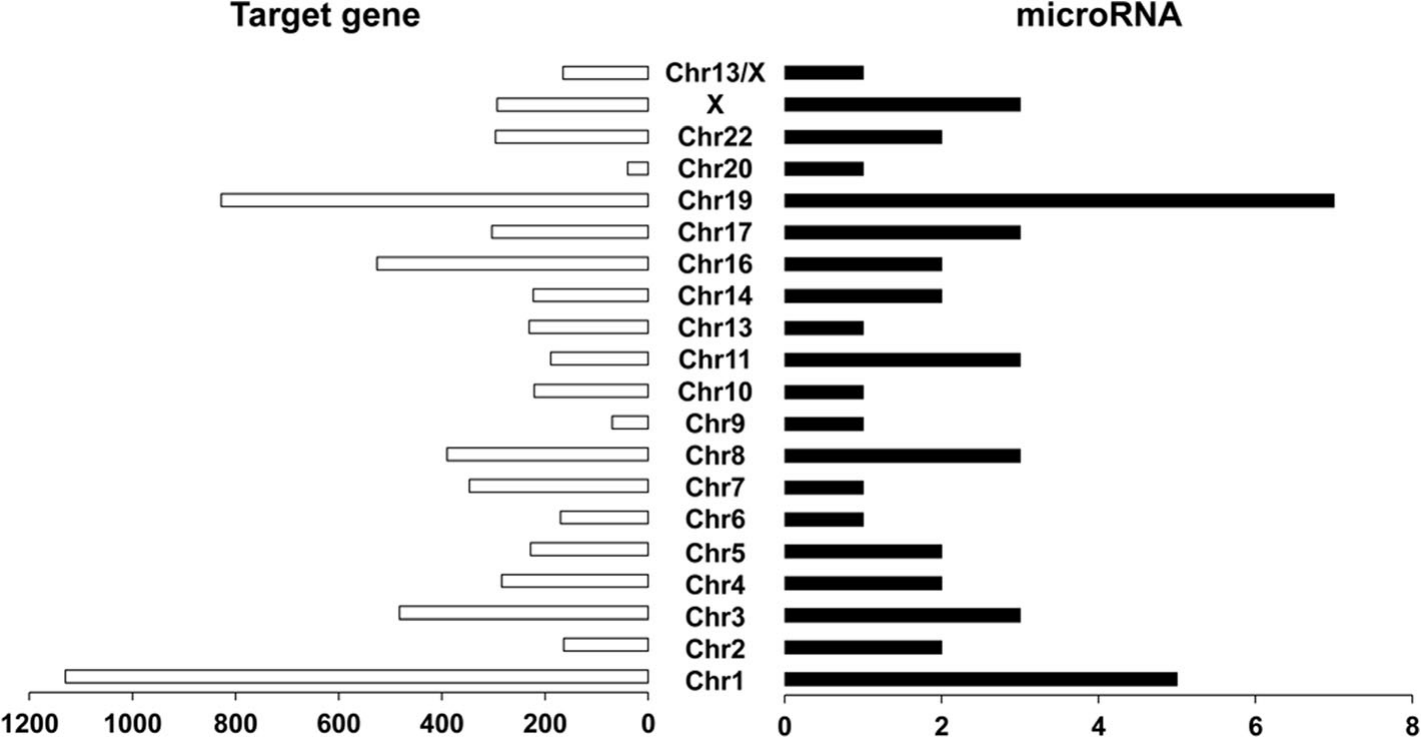
Chromosome loci of microRNAs and the number of target genes. The right column represents the number of differentially expressed microRNAs located in different chromosome. The left column represents the number of genes targeted by those miRNAs

**Fig. 4:**
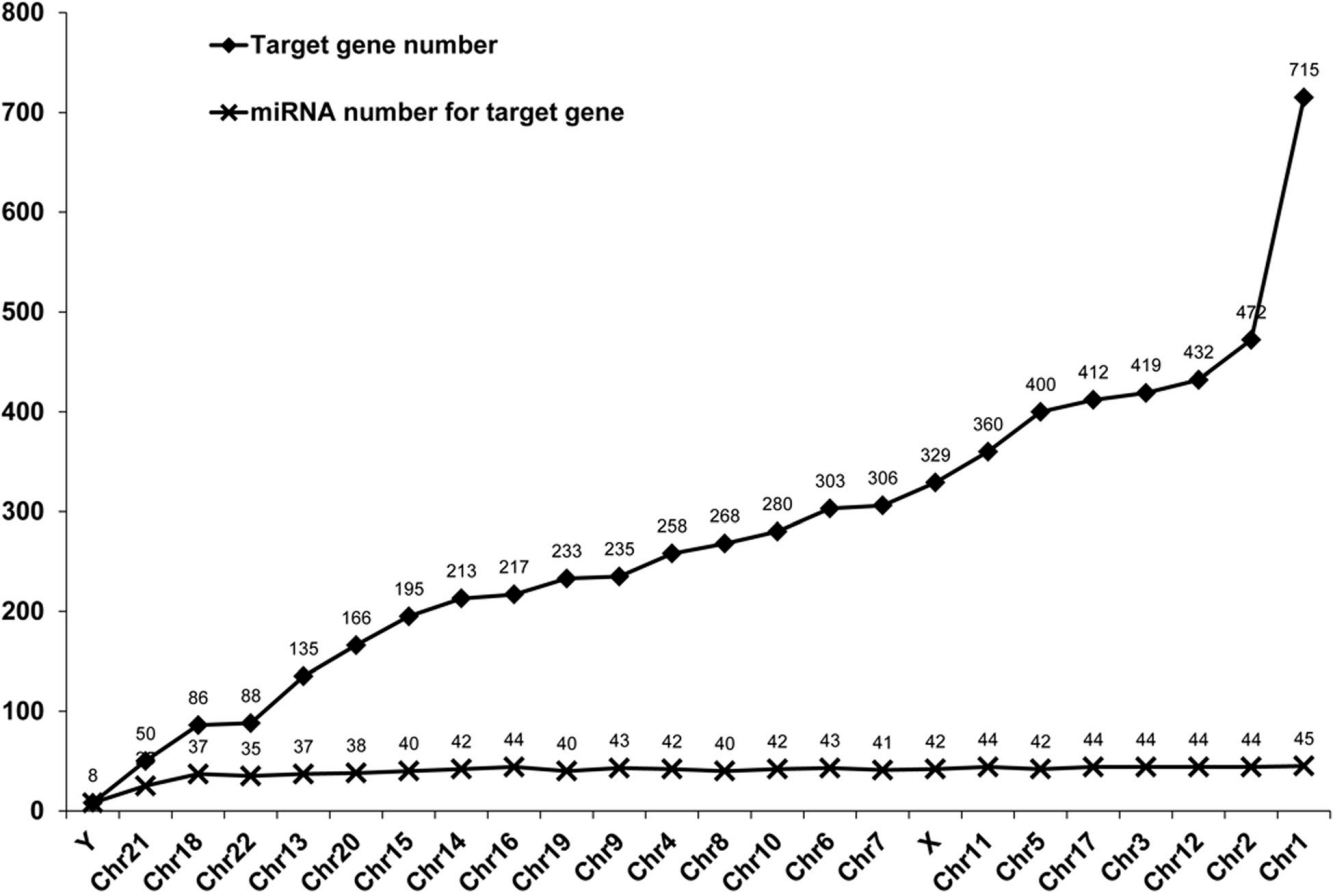
Chromosome loci of target genes and microRNA numbers with target genes. The number of target genes located in each chromosome and the number of miRNAs regulating those genes were shown

Target genes of the microRNAs were predicted by microRNAorg, PITA and TargetScan. Target genes that were simutaneously predicted by these three tools were selected (Fig. 3 and Fig. 4). In total, 6580 target genes were obtained (Additional file 1: Figure S1). We further screened out 47 microRNAs that had target genes (Tables 1 and 2). The number of up-regulated and down-regulated microRNAs that had target genes was almost equal (Table 1). Hsa-miR-765, hsa-miR-940, hsa-miR-1183, hsa-miR-557 and hsa-miR-939-5p had over 300 target genes. Hsa-miR-765 had the largest number of target genes (475) (Table 3).

**Table 3:** Chromosome loci and OMIM disease annotation of microRNA

MicroRNA	Chr_location	Number of target genes	MIM Number	MIM Title
hsa-miR-1182	1q42.2	145	#145500/ + 106,150	HYPERTENSION, ESSENTIAL/ ANGIOTENSINOGEN; AGT
hsa-miR-30e-5p	1p34.2	73	–	–
hsa-miR-557	1q24.2	306	*131210/ *182330/ #145500	SELECTIN E; SELE/ ATPase, Na+/K+ TRANSPORTING, BETA-1 POLYPEPTIDE; ATP1B1/ HYPERTENSION, ESSENTIAL
hsa-miR-765	1q23.1	475	–	–
hsa-miR-92b-3p	1q22	131	#151660	LIPODYSTROPHY, FAMILIAL PARTIAL, TYPE 2; FPLD2
hsa-miR-149-5p	2q37.3	153	–	–
hsa-miR-933	2q31.1	11	–	–
hsa-miR-1224-3p/5p	3q27.1	260	–	–
hsa-miR-198	3q13.33	169	–	–
hsa-miR-564	3p21.31	53	*160790	MYOSIN, LIGHT CHAIN 3, ALKALI, VENTRICULAR, SKELETAL, SLOW; MYL3
hsa-miR-574-5p	4	179	–	–
hsa-miR-575	4q21.22	105	–	–
hsa-miR-378a-3p	5q32	86	–	–
hsa-miR-874-3p	5q31.2	142	#614495/ *605775	PSEUDOHYPOALDOSTERONISM, TYPE IID; PHA2D/ KELCH-LIKE 3; KLHL3
hsa-miR-877-5p	6p21.33	170	–	–
hsa-miR-1183	7	347	–	–
hsa-miR-1234-3p	8	16	–	–
hsa-miR-30d-5p	8q24.22	69	–	–
hsa-miR-939-5p	8q24.3	305	#103900/ *608216	GLUCOCORTICOID-REMEDIABLE ALDOSTERONISM; GRA/ COMM DOMAIN-CONTAINING PROTEIN 5; COMMD5
hsa-miR-601	9q33.3	70	–	–
hsa-miR-936	10q25.1	221	*601568	ADDUCIN 3; ADD3
hsa-miR-1237-3p	11	129	–	–
hsa-miR-129-2-3p	11p11.2	26	#125853	DIABETES MELLITUS, NONINSULIN-DEPENDENT; NIDDM
hsa-miR-483-3p	11p15.5	34	#125852/ *191290	DIABETES MELLITUS, INSULIN-DEPENDENT, 2/ TYROSINE HYDROXYLASE; TH
hsa-miR-623	13q32.3	231	–	–
hsa-miR-1260a	14	134	–	–
hsa-miR-494-3p	14q32.31	89	–	–
hsa-miR-484	16p13.11	133	*603234/ #264800	ATP-BINDING CASSETTE, SUBFAMILY C, MEMBER 6; ABCC6/ PSEUDOXANTHOMA ELASTICUM; PXE
hsa-miR-940	16p13.3	393	#173900/ *610886/ *601313	POLYCYSTIC KIDNEY DISEASE 1; PKD1/ ESSENTIAL MEIOTIC ENDONUCLEASE 1, S. POMBE, HOMOLOG OF, 2; EME2/ POLYCYSTIN 1; PKD1
hsa-miR-144-3p	17q11.2	43	*163730/ #162200/ #145500	NITRIC OXIDE SYNTHASE 2A; NOS2A/ NEUROFIBROMATOSIS, TYPE I; NF1/ HYPERTENSION, ESSENTIAL
hsa-miR-22-3p	17p13.3	97	–	–
hsa-miR-636	17q25.1	163	–	–
hsa-miR-1238-3p	19	51	–	–
hsa-miR-1323	19q13.42	157	–	–
hsa-miR-371a-5p	19q13.42	183	–	–
hsa-miR-498	19q13.42	188	–	–
hsa-miR-516a-5p	19q13.42	12	–	–
hsa-miR-526b-5p	19q13.42	115	–	–
hsa-miR-769-3p	19q13.32	122	+ 107,741	APOLIPOPROTEIN E; APOE
hsa-miR-1825	20	40	–	–
hsa-miR-1281	22	12	–	–
hsa-miR-659-3p	22q13.1	284	–	–
hsa-miR-92a-3p	13q31.3/Xq26.2	165	–	–
hsa-miR-223-3p	Xq12	62	–	–
hsa-miR-361-5p	Xq21.2	92	–	–
hsa-miR-766-3p	Xq24	139	–	–

Chr_location, chromosome locus of microRNA. OMIM, Online Mendelian Inheritance in Man. * indicates that this record is a gene; # indicates that this is a descriptive record, often a phenotype, not a unique locus. + indicates that this record contains a description of the genes and phenotypes of known sequences

### Functional classification and pathway analysis of the target genes

The biological functions of the target genes were analyzed by GO. Most target genes were involved in the regulation of transcription, signal transduction, organismal development and cell adhesion (Additional file 2: Figure S2A). The number of target genes in cytoplasm and nucleus was almost equal (Additional file 2: Figure S2B). Over half of the target genes (53.8%) had protein binding function, and about 40% of the genes may play roles in zinc-ion binding (17.5%) or metal-ion binding (23.9%) (Additional file 2: Figure S2C).

**Fig. 5:**
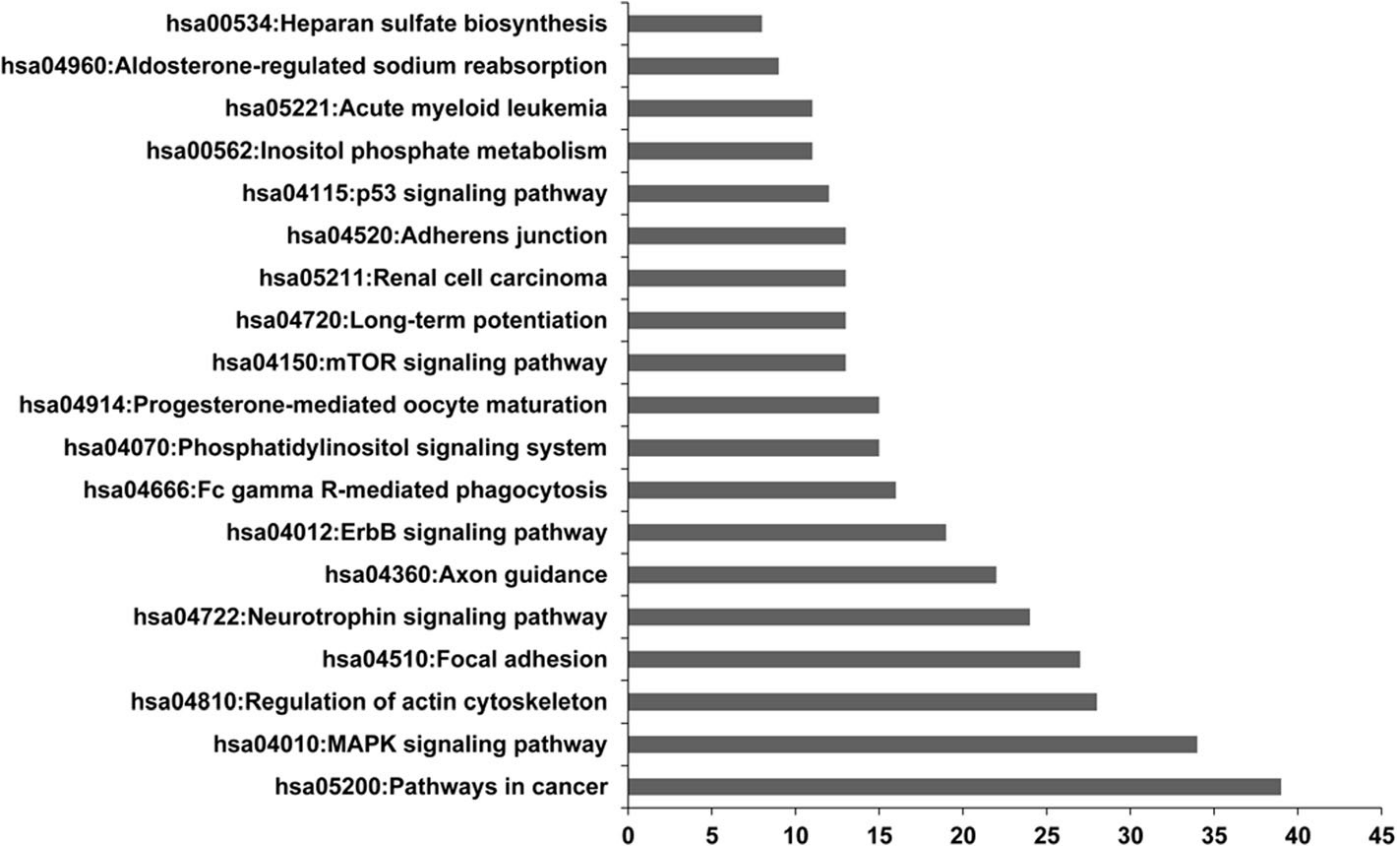
Pathway analysis that target genes are involved in. The x-axis represents the number of genes involved in corresponding pathway. The y-axis represents names of the top 19 pathways

The target genes were classified into different functional groups based on GO-terms. It is well worth noting that most of genes were enriched in ion binding group (ES = 3.46) and transcription regulation group (ES = 3.44). Target genes involved in cell fraction and cell adhesion were enriched with an enrichment score of 5.55 and 4.33, respectively. In addition, pathway analysis was performed to analyze the signal pathways that target genes or microRNAs may participate in. We found that the target genes were mainly involved in cancer pathways, MAPK pathway, regulation of actin cytoskeleton and focal adhesion (Fig. 5).

### Cytogenetic location and OMIM annotation of the microRNAs

Cytogenetic location of the 47 microRNA candidates were analyzed. Most of them were located at chromosome 19 (40 microRNAs) and chromosome 1 (45 microRNAs) (Table 3). No microRNA was found on chromosome 12, 15, 18 or Y. Moreover, OMIM records of the patients with essential hypertension were evaluated. Totally, 13 OMIM records about essential hypertension were obtained. The records illustrated the relationship between microRNAs and disease status (Table 3). Hsa-miR-1182, hsa-miR-557 and hsa-miR-144-3p showed a direct correlation with essential hypertension (OMIM ID #145500). Moreover, miR-92b-3p, miR-939-5p, and miR-769-3p were annotated with lipodystrophy, glucocorticoid and apolipoprotein E, which are hypertension-related indicators.

### Verification of the potential microRNA markers and microRNA-gene interaction

**Fig. 6:**
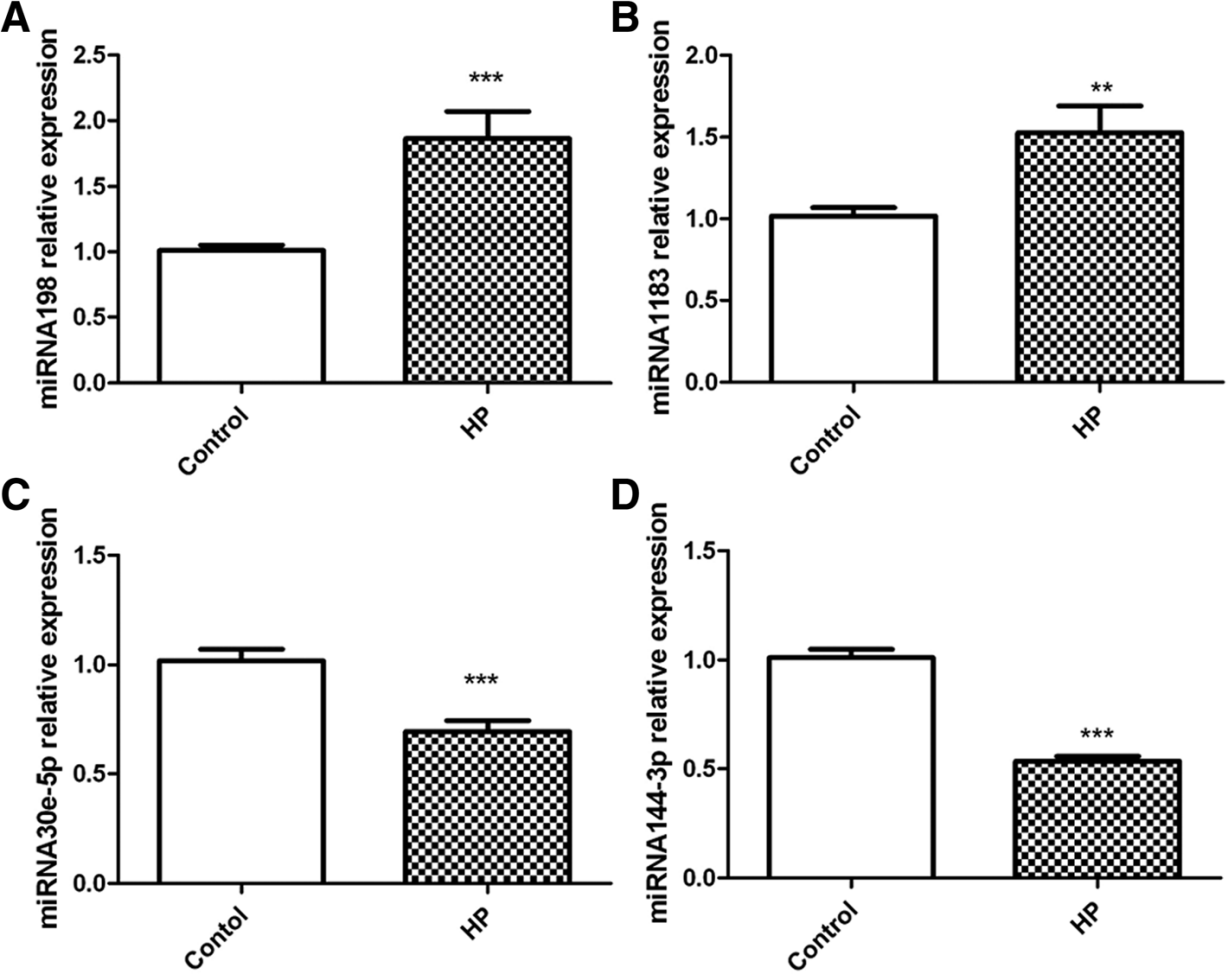
Quantitative expression analysis of differentially expressed microRNA by qRT-PCR. Control: healthy samples; HP: essential hypertension samples. Relative expressions of (**a**) miRNA198, (**b**) miRNA1183, (**c**) miRNA30e-5p, and (**d**) miRNA144-3p were shown. ***: *p*-value< 0.01, **: *p*-value< 0.05

**Fig. 7:**
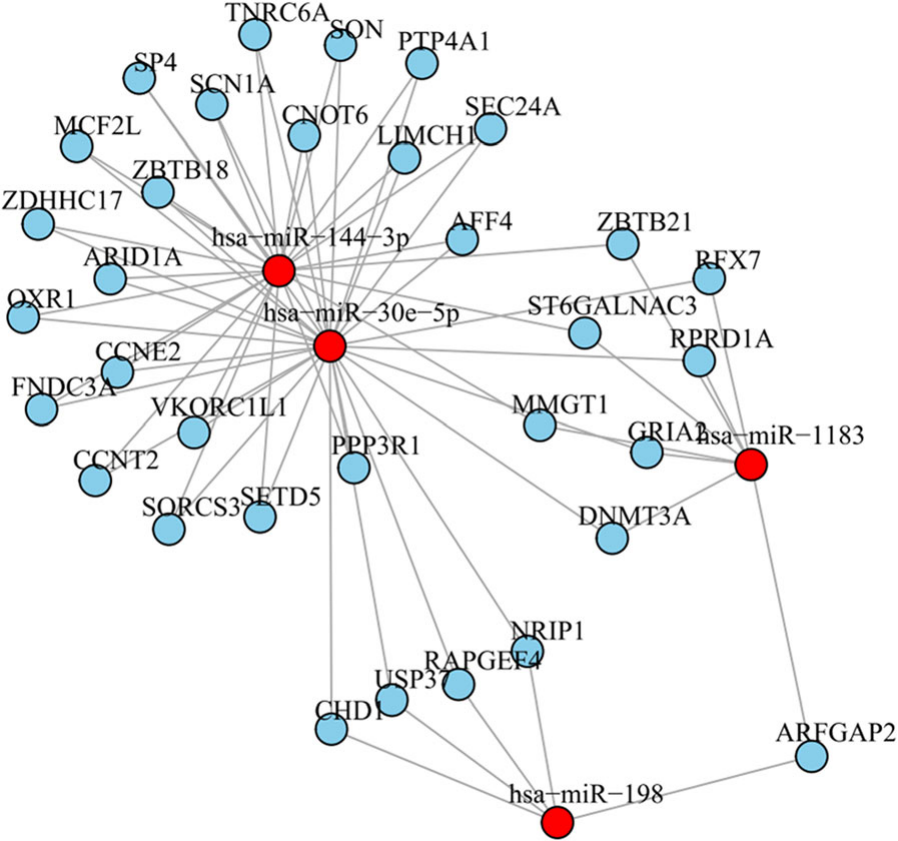
Interaction network of microRNAs and target genes. Red dot represents the four microRNAs of interest that show the most significant expression changes in essential hypertension group. Light blue dot represents target genes of microRNAs. Grey lines represent the interaction between the microRNAs and the target genes

Four microRNA candidates with significant expression change were verified by qPCR. MiR-198 and miR-1183 showed a higher expression in patients with essential hypertension while miR-30e-5p and miR-144-3p showed lower expression (Fig. 6). Fold change of microRNAs (miR-198, miR-1183, miR-30e-5p and miR-144-3p) measured by qPCR was in accordance with the results of microRNA array (Table 4). In order to explore the relationship between microRNAs and target genes, a microRNA-gene interaction network was constructed (Fig. 7). Target genes of the four microRNAs were selected with a tougher criteria. Target genes were graded using miRBase database firstly and then the genes with scores of more than 80 were selected. Altogether, 33 target genes were obtained. MiR-144-3p shared 21 target genes with miR-30e-5p, while it didn’t share any target gene with miR-198 (Fig. 7). MiR-1183 and miR-198 both regulated ARFGAP2. MiR-1183 and miR-144-3p both targeted MMGT1, ST6GALNAC3 and ZBTB21.

**Table 4:** Fold change of selected microRNAs

miRNA	Microarray fold change	qPCR fold change	Change
miRNA198	42.16	1.87	Up
miRNA1183	44.41	1.53	Up
miRNA30e-5p	25.00	1.45	Down
miRNA144-3p	19.41	1.87	Down

## Discussion

In this study, we identified 257 differentially expressed microRNAs from 4 Uyghur patients (from Xinjiang, China) with essential hypertension and constructed a candidate microRNA biomarker set of 47 microRNAs. To the best of our knowledge, this study is the first report to screen microRNA markers of essential hypertension in Uyghur population of Xinjiang, China. Our results add new information to the potential microRNA markers of essential hypertension.

Among the 47 microRNAs, the number of up-regulated and down-regulated microRNAs was almost equal. However, we found that the expression of up-regulated microRNAs changed more significantly. Compared with the microRNA profiles of hypertension reported by previous studies [2–4], many new microRNAs were identified in this study. The difference may be due to the complexity of hypertension or the differences in sampling population or methods [2, 4].

Previous studies have shown that essential hypertension may be caused by genetic factors [1, 2]. Here, we found that the microRNAs were mostly located on chromosome 1 and chromosome 19. Five miRNAs located at different loci of chromosome 1 and seven miRNAs located at chromosome 19, five of which located at the same locus (19q13.42). Further analysis through the OMIM database revealed that miR-1182 was annotated with angiotensinogen, a protein related to essential hypertension [4, 8]. Moreover, miR-557 was related to ATPase and Na^+^/K^+^ transport, which play important roles in the blood pressure regulation [12, 13]. miR-769-3p was annotated with apolipoprotein, a molecule that is closely related to cardiac diseases [14]. These results demonstrate that the idenfied microRNAs are related with the metabolic processes of essential hypertension, indicating that these microRNAs may be involved in the development of essential hypertension.

Target genes were clustered into 8 groups by functional annotation and most of them were enriched in metal ion binding and transcription regulation (Table 5). Abnormal Ca^2+^/Na^+^ is one of the common phenomena in hypertension and cardiovascular diseases [12]. Annotation analysis both in microRNAs and target genes showed a tendency in ATP and mineral ions metabolism. This suggests that these microRNAs may be used as potential diagnostic markers. Further studies on the role and mechanism of microRNAs in essential hypertension are needed.

**Table 5:** GO classification of target genes

Enrichment Score	Term	Count	%	P-value
5.55	GO:0000267~cell fraction	117	8.93	2.35E-06
	GO:0005624~membrane fraction	93	7.10	2.56E-06
	GO:0005626~insoluble fraction	95	7.25	3.76E-06
4.90	GO:0030424~axon	31	2.37	6.00E-07
	GO:0043005~neuron projection	49	3.74	3.38E-06
	GO:0042995~cell projection	72	5.50	9.96E-04
4.33	GO:0007156~homophilic cell adhesion	32	2.44	3.32E-09
	GO:0016337~cell-cell adhesion	40	3.05	5.08E-05
	cell adhesion	50	3.82	1.66E-04
	GO:0007155~cell adhesion	72	5.50	0.002747
	GO:0022610~biological adhesion	72	5.50	0.002857
3.46	GO:0046872~metal ion binding	367	28.02	1.88E-05
	zinc-finger	160	12.21	2.68E-05
	GO:0043169~cation binding	367	28.02	4.67E-05
	GO:0043167~ion binding	370	28.24	7.72E-05
	zinc	188	14.35	4.53E-04
	metal-binding	243	18.55	9.94E-04
3.44	transcription regulation	195	14.89	3.24E-07
	Transcription	198	15.11	3.92E-07
	nucleus	364	27.79	7.14E-07
	GO:0030528~transcription regulator activity	152	11.60	6.06E-05
	GO:0045449~regulation of transcription	234	17.86	2.32E-04
	GO:0006350~transcription	191	14.58	6.37E-04
	GO:0043565~sequence-specific DNA binding	65	4.96	0.002591
	GO:0003700~transcription factor activity	95	7.25	0.004676
3.22	GO:0014069~postsynaptic density	18	1.37	6.30E-06
	GO:0045202~synapse	48	3.66	2.05E-05
	GO:0030054~cell junction	61	4.66	9.09E-05
	cell junction	47	3.59	3.04E-04
	synapse	29	2.21	5.96E-04
	GO:0044456~synapse part	29	2.21	0.00823
3.20	ubl conjugation pathway	62	4.73	1.05E-05
	GO:0051603~proteolysis involved in cellular protein catabolic process	70	5.34	1.00E-04
	GO:0044257~cellular protein catabolic process	70	5.34	1.15E-04
	GO:0043632~modification-dependent macromolecule catabolic process	66	5.04	2.46E-04
	GO:0019941~modification-dependent protein catabolic process	66	5.04	2.46E-04
	GO:0030163~protein catabolic process	70	5.34	2.89E-04
	GO:0006511~ubiquitin-dependent protein catabolic process	34	2.60	3.62E-04
	GO:0044265~cellular macromolecule catabolic process	74	5.65	0.002887
3.12	kinase	80	6.11	2.92E-06
	nucleotide-binding	159	12.14	1.50E-05
	transferase	132	10.08	7.49E-05
	atp-binding	125	9.54	1.39E-04
	GO:0006796~phosphate metabolic process	102	7.79	1.62E-04
	GO:0006793~phosphorus metabolic process	102	7.79	1.62E-04
	GO:0006468~protein amino acid phosphorylation	73	5.57	4.73E-04
	GO:0017076~purine nucleotide binding	178	13.59	8.59E-04
	GO:0016310~phosphorylation	83	6.34	9.76E-04
	GO:0032553~ribonucleotide binding	170	12.98	0.001314
	GO:0032555~purine ribonucleotide binding	170	12.98	0.001314
	serine/threonine-protein kinase	43	3.28	0.001333
	GO:0001883~purine nucleoside binding	148	11.30	0.003017
	GO:0030554~adenyl nucleotide binding	146	11.15	0.003068
	GO:0000166~nucleotide binding	199	15.19	0.0037
	GO:0001882~nucleoside binding	148	11.30	0.003985
	GO:0005524~ATP binding	137	10.46	0.004062
	GO:0032559~adenyl ribonucleotide binding	138	10.53	0.004694

In our study, target genes related to cell adhesion and junction were enriched into two clusters. Researchers have found that miR-92a may play a role in mediating cell-to-cell communication [15–17]. The miR-1 may affect the genes of gap-junction channels [12, 18]. Our results reveal that microRNAs may participate in regulating cell communications in essential hypertension. Pathway analysis also supported their role in cell to cell communication. Genes in this pathway are involved in focal adhesion and regulation of actin cytoskeleton and adherence junction. However, sources of microRNAs in plasma are various. Apoptotic, necrotic cell death and active secretion all result in detectable microRNAs in plasma [19].

MiR-198 and miR-1183 were the two most significantly up-regulated microRNAs according to the microarray results, while miR-30e-5p and miR-144-3p were the two most significantly down-regulated microRNAs. QPCR further confirmed the microarray results. However, the fold change of microRNA expression measured by qPCR was not as significant as that by microarray. Li et al. found that miR-1183 was a potential diagnostic marker for rheumatic heart disease [20]. High expression level of miR-198 is reported in acute coronary syndromes [21]. miR-30e-5p is found to suppress the expression of hypertension related gene ADRA2A in human endothelial cells [22]. miR-144-3p regulates cholesterol homeostasis and is identified as a potential therapeutic target of atherosclerosis and acute myocardial infarction [23]. However, how are these four microRNAs involved in essential hypertension are unclear. The significant changes of their expression in this research may pave a way for further study.

Interaction analysis can greatly help us to understand the relationship of microRNAs with target genes. One microRNA may regulate multiple genes, and one gene might be regulated by different microRNAs [3–5]. In this study, the target genes of miR-198, miR-1183, miR-30e-5p and miR-144-3p were predicted and a microRNA-gene interaction network was constructed. Totally, 33 target genes were obtained. However, functional analysis and validation of the target genes are not performed and needs further study.

This study has some limitations. First, this study focused on Uyghur subjects from Xinjiang, China. Thus, the results of this study may lack representativeness for the general population. Second, the sample size was relatively small. Third, the number of differentially expressed microRNAs validated by qPCR was small. Fourth, the function of the 33 target genes of miR-1183, miR198, miR144-3p and miR-30e-5p was not further analyzed. Further studies are warranted.

In conclusion, we constructed a reference microRNA pool of essential hypertension and obtained a set of circulating microRNAs, which may be used as potential biomarkers for essential hypertension. These microRNAs showed a remarkable correlation with mineral ion metabolism and cellular communication. Four microRNAs (miR-1183, miR-198, miR-30e-5p and miR-144-3p) that showed most prominent change were further verified. A regulatory network between these microRNAs and their target genes was also constructed, which may provide experimental evidence for further study of the mechanism of essential hypertension.

## Additional files

Additional file 1: **Figure S1.** Target genes prediction by three microRNA databases**.** Targetscan (http://www.targetscan.org/), microRNAorg (http://www.microrna.org/), and, pita (https://genie.weizmann.ac.il/pubs/mir07/) was used (TIF 99 kb)

Additional file 2: **Figure S2.** GO analysis**.** (A) Target gene coverage of GO biological process term. (B) Target gene coverage of GO cellular component terms. (C) Target gene coverage of GO molecular function terms (TIF 503 kb)

## Abbreviations

GO: Gene Ontology
HGNC: Hugo Gene Nomenclature Committee
KEGG: Kyoto Encyclopedia of Genes and Genomes
OMIM: Online Mendelian Inheritance in Man
qPCR: Quantitative Real-time PCR
