# Supplementary figures and images for: Screening of differentially expressed microRNAs of essential hypertension in Uyghur population
Source: Lipids Health Dis. 2019 Apr 11;18:98. doi: 10.1186/s12944-019-1028-1 (PMC6460779; doi:10.1186/s12944-019-1028-1)

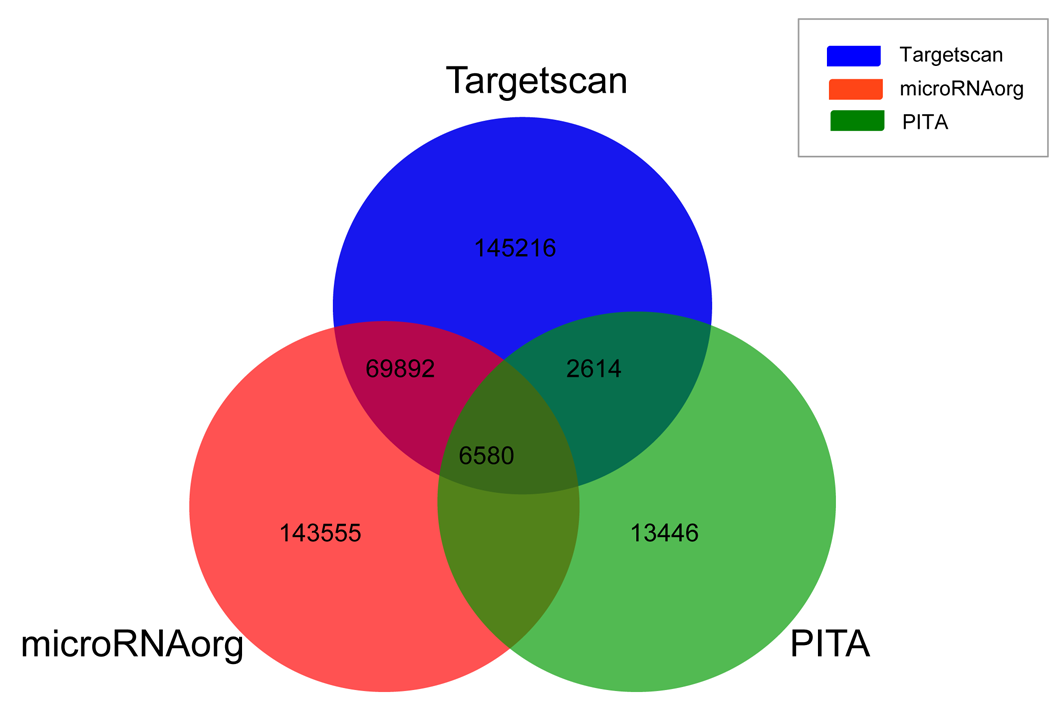

Supplement: Supplementary file 1 — Figure S1. Target genes prediction by three microRNA databases. Targetscan (http://www.targetscan.org/), microRNAorg (http://www.microrna.org/), and, pita (https://genie.weizmann.ac.il/pubs/mir07/) was used (TIF 99 kb) [file 12944_2019_1028_MOESM1_ESM.tif]

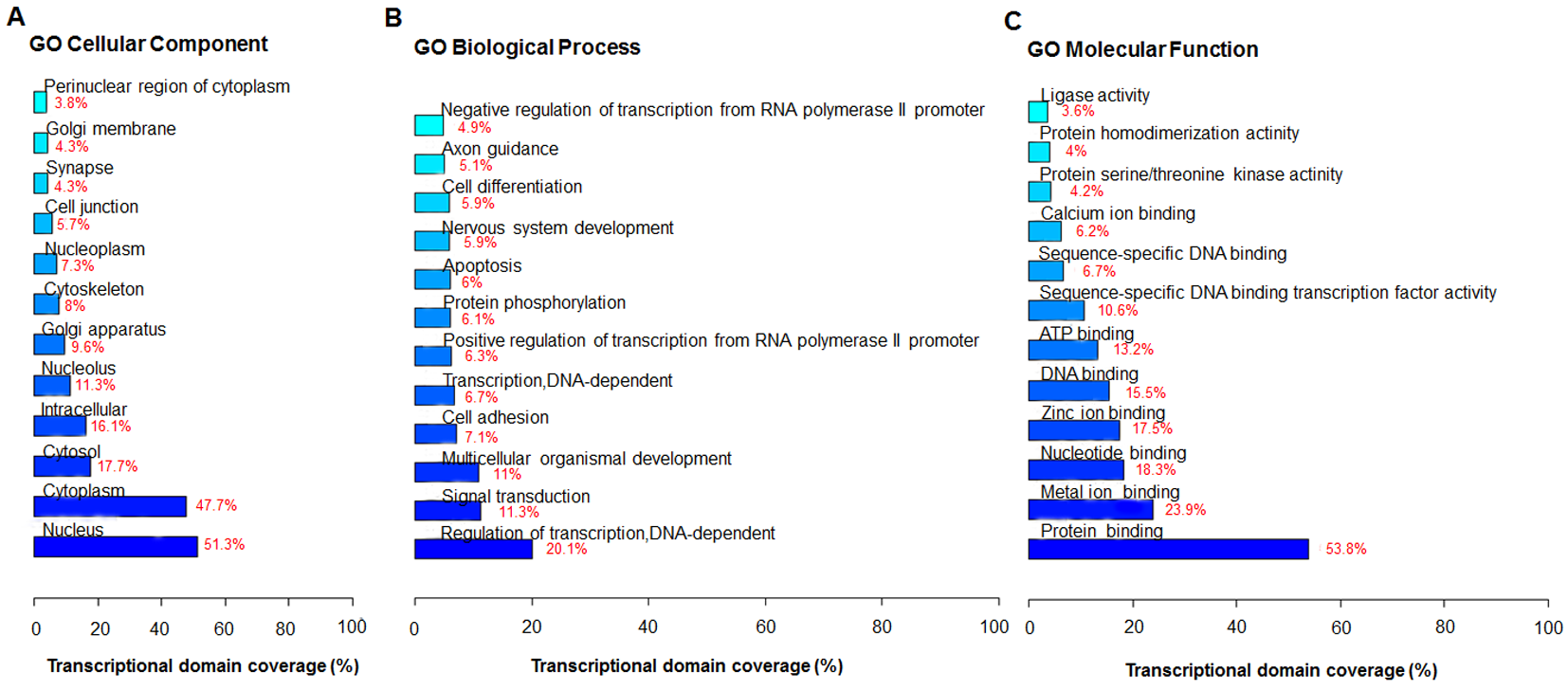

Supplement: Supplementary file 2 — Figure S2. GO analysis. (A) Target gene coverage of GO biological process term. (B) Target gene coverage of GO cellular component terms. (C) Target gene coverage of GO molecular function terms (TIF 503 kb) [file 12944_2019_1028_MOESM2_ESM.tif]
